# Epidemiological Implications of HIV-Hepatitis C Co-Infection in South and Southeast Asia

**DOI:** 10.1007/s11904-014-0206-z

**Published:** 2014-03-29

**Authors:** Shaodong Ye, Lin Pang, Xiaochun Wang, Zhongfu Liu

**Affiliations:** National Center for AIDS/STD Control and Prevention, China Center for Disease Control and Prevention, 155 Changbai Road, Changping District, Beijing, 102206 People’s Republic of China

**Keywords:** HIV, Hepatitis C, HIV-HCV Co-infection, Risk factors, South and Southeast Asia, Injection drug use, Blood donor, Prevalence, Global epidemic, HIV-positive, Southeast Asia, South Asia

## Abstract

We sought to profile the epidemiological implication of human immunodeficiency virus (HIV) and hepatitis C virus (HCV) co-infection from South and Southeast Asia by reviewing original studies reporting prevalence of HIV-HCV co-infection and their risk factors. Thirteen papers cited in the PubMed database and published in 2012 and 2013 were reviewed. The overall HCV co-infection prevalence ranged broadly from 1.2 % to 98.5 % among HIV-positive people in South and Southeast Asia. Among HCV seropositive blood donors in Nepal, 5.75 % had HIV co-infection. Injecting drug use (IDU) was one of the key risk factors of co-infection, with HCV infection reaching 89.8 % and 98.5 % among HIV-positive injecting drug users in Vietnam. The most recent data from South and Southeast Asia suggest the urgency of implementation of comprehensive prevention and control strategies of HIV-HCV co-infection.

## Introduction

The 150 million people chronically infected with hepatitis C virus (HCV) suggest a 2.3 % global prevalence, higher even than the 35.3 million people living with human immunodeficiency virus (HIV), about 0.54 % prevalence. However, death rates are inverted with about 350,000 deaths/year from HCV-related liver diseases to 1.6 million deaths/year due to HIV/AIDS, as estimated in 2013 by the World Health Organization (WHO) and the Joint United Nations Program on HIV/AIDS (UNAIDS) [1•, 2•].

Given sexual, blood, and perinatal transmission routes, HIV and HCV can be expected to be transmitted in similar at-risk populations. Reports are variable, but reports range from 7-30 % HCV co-infection among HIV patients [3–7] and 6-10 % HIV co-infection among HCV patients [5, 8••]. Co-infections influence each other and affect the pathogenesis and even causes of death for HIV-HCV infected persons, complicating both treatment and even prevention strategies [9, 10].

The estimated number of HIV cases in 19 countries of South and Southeast Asia ranks second just after Sub-Saharan Africa [2•]. Many of these nations have made substantial efforts to combat HIV/AIDS with a variety of education and prevention strategies, including efforts towards stigma reduction, interventions targeting commercial sex workers (CSW), injection drug users (IDU), expanded antiretroviral treatment (ART), and ensuring a safe blood supply, among others.

HCV infections are serious in these same countries, too, due to blood transfusions and IDU [11, 12]. With the expansion of the HIV epidemic, increased awareness, and better diagnostic capacities, more HIV-HCV co-infections have been reported with increasing frequency [11, 13–15, 16••, 17••, 18••]. Many studies have introduced these co-epidemics, analyzed the risk factors, discussed treatment, and suggested strategies of prevention and control in individual countries. While a few reviews included South and Southeast Asia [19, 20], there has been little targeted attention on HIV-HCV co-infection specifically for South and Southeast Asia.

Our paper aims uses recently published literature to provide a profile of the epidemiological circumstances for HIV-HCV prevalence and risk factors in South and Southeast Asia. We hope this stimulates increased attention to both prevention and clinical care for these highly populous nations.

## Methods

### Search Strategy

The literature on HIV-HCV co-infection published in English in 2012 and 2013 was searched with the PubMed database. Keywords used in the database search included: [(hepatitis C or HCV) AND (HIV OR AIDS) AND (2012 OR 2013)] AND [(South Asia or Southeast Asia) or (individual country names of South and Southeast Asia)] (Table 1): All publications were sorted with an Endnote® file (Endnote X4®, Thomson Reuters, San Francisco, CA: Full texts were obtained from ProQuest® Health & Medical through the Chinese Center for Disease Control and Prevention.

**Table 1:** Countries of South and Southeast Asia included in this literature-based review

South Asian countries	Southeast Asian countries
• Bangladesh • Pakistan • Nepal • Maldives • Republic of India • Sri Lanka • Bhutan • Afghanistan	• Brunei • Thailand • East Timor • Vietnam • Indonesia • Malaysia • Philippines • Myanmar • Cambodia • Singapore • Laos

### Study Selection

The following criteria were used to select studies: original study on HIV-HCV co-infection; available prevalence rate of co-infection and/or odds ratio (OR) for putative risk factors; eligible countries of South and Southeast Asia. All titles and abstracts were reviewed to decide the inclusion of the eligible papers. Full texts were also reviewed whenever necessary, to check the data details and to judge the relevance of the epidemiological observations of HIV-HCV co-infection.

## Results

### Result of Literature Search

A total of 23 and 39 papers were found in South and Southeast Asia in 2012 and 2013, respectively, of which 54 were unique (8 duplicates). A further 10 papers were excluded because they were not actually studies in South and Southeast Asia. Nine more papers were excluded due to containing only HIV or HCV information. Reviews of the abstracts and full texts of the remaining 35 papers led to further exclusion of 22 papers which had no data regarding HIV-HCV co-infection. Thus a total of 13 papers met the review needs [8••, 16••, 17••, 18••, 21••, 22••, 23••, 24••, 25••, 26••, 27••, 28••, 29••].

### Description of Studies

Among the 13 studies shown in Table 2, two South Asian studies were conducted in India and three in Nepal. Among eight Southeast Asian studies, two were in Vietnam, one in Cambodia, one in Myanmar, two in Thailand, and two in Indonesia. Except for one retrospective cohort and one case control study, 11 were designed as cross-sectional studies. The sample size of the studies ranged widely from 126 to 16,124. Eight studies used HIV-positive samples to assess HCV prevalence including one study among HIV-infected IDUs. Four studies used high risk populations, including one study among CSWs, one study among IDU inmates, and one among both CSWs and IDUs. The last study was conducted among blood donors who were HCV seropositive.

**Table 2:** Characteristics of 13 HIV-HCV co-infection studies in South and Southeast Asia

Publication	Country	Study design	Samples	HIV-HCV%	Risk factors
Nagmoti et al. (2012) [16••]	India	cross sectional	16,124 serum samples from suspected patients	3.52	
Praseeda et al. (2013) [25••]	India	cross-sectional	250 CSWs	1.2	
Shrestha et al. (2012) [8••]	Nepal	cross-sectional	139 HCV seropositive blood donors	5.75	
Barnawal et al. (2013) [21••]	Nepal	cross sectional	313 HIV patients	42	Male gender (p < 0.001) and IDU (p < 0.001), lower CD4 counts (p <0.05)
Poudel et al. (2013) [24••]	Nepal	cross-sectional	319 HIV-positive	43.3	96.2 % (125/130) HCV co-infection among HIV-positive IDUs
Dunford et al. (2012) [22••]	Vietnam	cross sectional	8654 high risk individuals	89.8 % HCV of the HBV-HIV co-infected IDUs, and 40 % HCV of HBV-HIV co-infected CSWs	
Sereno et al. (2012) [27••]	Vietnam	cross-sectional	HIV infected drug users	98.5	
Goyet et al. (2013) [23••]	Cambodia	case-control	44 HCV/HIV co-infected patients and 160 HIV mono-infected patients without HCV antibodies		age older than 50 years (OR 5.4, 95 % CI:1.5-19.6), the exposure to multiple parenteral infusions before the year 2000 (OR 3.4, 95 % CI 1.5-7.6), surgery (OR 2.6, 95 % CI 1.2-5.7) and fibroscopy (OR 2.4, 95 % CI 1.0-5.7)
Zaw et al. (2013) [17••]	Myanmar	cross-sectional	11,032 HIV-infected patients	5.3	IDU (p < 0.05)
Than et al. (2012) [28••]	Thailand	retrospective cohort	57 HIV-infected patients with HCV and 114 HIV-infected patients without HCV		HCV co-infection does not appear to affect HIV morbidity, mortality or treatment responses to ART during follow-up time of 2.9 (1.2-9.8) years as only 7 % (4/57) HCV co-infected patients were treated for HCV infection.
Tsuchiya et al. (2013) [29••]	Thailand	cross-sectional	700 HAART-naive HIV-infected patients	3.3	HCV co-infection associated probably with higher mortality of HIV aHRs = 1.90 (95 % CI: 0.98-3.69)
Anggorowat et al. (2012) [18••]	Indonesia	cross sectional	126 HIV patients	34.1	IDU (OR: 26.52; 95 % CI: 3.52-199.54) and alanine aminotransferase >/=40 IU/L (OR: 6.36; 95 % CI: 1.23-32.89)
Prasetyo et al. (2013) [26••]	Indonesia	cross-sectional	375 drug abuser inmates	4	

HIV: Human Immunodeficiency Virus; HCV: hepatitis C; IDU: injecting drug use; CSW: commercial sex work; ART: antiretroviral treatment; OR: odds ratio; CI: confidence interval; aHRs: adjusted hazard ratios; IU/L: International Unit/litre

## HIV-HCV Co-infection Prevalence

The overall HIV-HCV co-infection prevalence among HIV-positive persons ranged broadly from 1.2 % to 98.5 % in South and Southeast Asia, as shown in Table 2. In six studies testing HIV-positive samples, the HIV-HCV co-infection rate was relatively higher in Indonesia, Nepal and Vietnam, ranging between 34.1 % and 98.5 % [18••, 21••, 22••, 24••, 27••]. Particularly in Vietnam, it reached 89.8 % and 98.5 % among HIV-positive IDUs [22••, 27••]. In Myanmar and Thailand, the HIV-HCV co-infection rate among HIV-infected persons was comparatively lower than in Indonesia, Nepal, and Vietnam, 5.3 % [17••] and 3.3 % [29••] respectively. If the target population was persons with high risk behaviors, such as CSWs in India or IDU inmates in Indonesia, the co-infection prevalence were lower, about 1.2 ~ 4 % [16••, 25••, 26••]. The only study among HCV seropositive blood donors from Nepal published in 2012 showed 5.75 % HIV-HCV co-infection rate [8••].

## Risk Factors Associated with HIV-HCV Co-infection

Five cross-sectional studies were conducted among people with obvious high risk behavior, including one study among IDU inmates [26••], one study among HIV-positive IDUs [27••], one study among CSWs [25••], one study among both IDUs and CSWs [22••], and one study among HCV-positive blood donors [8••]. Five studies estimated the role of risk factors associated with HIV-HCV co-infection. Four cross-sectional studies strongly indicated IDU as the highest risk factor with a high proportion of co-infection [24••], suggested that results were not due to chance alone, [17••, 21••] and reported strong association in odds ratios [18••]. The only case-control study did not find an association with IDU, rather showing associations with age older than 50 years (OR 5.4, 95 % confidence interval (CI):1.5-19.6), the exposure to multiple parenteral infusions before the year 2000 (OR 3.4, 95 % CI 1.5-7.6), surgery (OR 2.6, 95 % CI 1.2-5.7), and fiber optic scoping (OR 2.4, 95 % CI 1.0-5.7) [23••]. Other risk factors associated with HIV-HCV co-infection were male sex (p < 0.001), lower CD4+ cell counts (p < 0.05) [21••], and alanine aminotransferase >40 IU/L (OR: 6.36; 95 % CI: 1.2-32.9) [18••].

Two studies examined the association of HIV-HCV co-infection with other outcomes, but few associations were significant; a probable higher mortality of HIV was noted with HCV co-infection (adjusted hazard ratios [aHRs] = 1.90; 95 % CI: 0.98-3.7) in one cross-sectional study [29••]. One retrospective cohort study suggested that HIV-HCV co-infection had no impact on morbidity, mortality, or treatment responses to ART, as there were HIV patient deaths during 2.9 years of follow-up, although only 7 % of HIV-HCV co-infected patients were treated for HCV infection in the case group [28••]. One study provided HIV-HCV co-infection rate but did not analyze risk factors [16••] (Table 2):

## Discussion

HIV and HCV are in completely different virus families, yet both are transmitted through bodily fluids and blood; both viruses can infect patients for years before symptoms are manifested [8••]. Given the attention given to HIV, it is more likely that HCV will be neglected; such neglect can result in irreversible hepatocirrhosis and hepatocarcinoma [1•]. Since 1985, HIV screening has been suggested for the blood supply and for persons at risk, with supportive public policies typically supporting this strategy. In contrast, HCV has commanded far less attention and public policies have lagged the need. We think the current literature does a good job alerting the public as to the seriousness of HCV and HIV-HCV joint infection, promoting screening and treatment policies based on a strategy to improve HCV medical care and prevention.

HIV-HCV co-infection publications suggested wide prevalence variability, from 1.2 % to 98.5 % HIV-HCV co-infection in South and Southeast Asia, notably higher among IDUs (34.1-98.5 %). The multivariable analyses in these cross-sectional studies confirm IDU as the major risk factor in this region, both for HIV-HCV co-infection, and for HIV and HCV independently. However, the case-control study in Cambodia suggested multiple parenteral infusions before the year 2000, surgery, and fibroscopy to be the principal risk factors, suggesting diversity between population samples [23••]. The lack of impact of HIV-HCV co-infection on morbidity, mortality or treatment responses to ART of HIV patients in two studies [30, 31] clashes with findings from other studies worldwide and may reflect methodological limitations of sample size and length of follow-up.

Most countries in South and Southeast Asia are not yet economically developed and there are strong forces towards increasing high risk behaviors such as IDU and CSW. Basic medical services are insufficient and often very expensive for poor people. Furthermore, HIV prevention and control programs are often weak with inadequate attention and funding support from national and local governments, even though political commitments to Millennium Development Goals (MDGs) have been declared.

The serious epidemics of HIV-HCV co-infection should be emphasized in the prevention and treatment of both diseases in Asia as there was less published research and fewer evidence prevention strategies compared to Western nations. Co-infection may accelerate the clinical progress to both diseases [13, 31–33] and treatment success for one disease is undermined when the other disease is neglected. Governments have paid greater attention to HIV prevention and control and typically provide free ARV treatment for HIV, but the high expenses of interferon and ribavirin for HCV treatment is still a great challenge which prohibits the access of this clinical service, especially in these resource limited settings of South and Southeast countries [28••]. These studies suggest national policies, strategies, and guidelines on HIV-HCV co-infection control should be revisited, including other blood-borne infections, such as hepatitis B virus, GB virus C (GBV-C), and others [23••, 27••]. Novel therapies are emerging quickly for HCV disease and countries must prepare for the increased opportunities for HCV management that will be emerging.

It is clear that HCV screening should be conducted as soon as an HIV-positive test is confirmed. This permits co-infection to be treated appropriately, quality care provided, and further disease progress prevented [17••, 23••, 24••]. ART-naïve HIV-infected patients may have tenofovir-based regimens given preferentially, given its impact on both HIV and HCV, for co-infected persons [29••]. Some unique communities, like prisons, are recommended to pay added attention to screening, prevention, and control of both infections and co-infection among inmates who used or continue to use drugs [26••].

Needle and syringe exchanges, opioid substitution therapy (especially with methadone maintenance or buprenorphine), and condom use are all advisable for persons who use drugs, to modulate risk from body fluid and blood-borne diseases. The sterilization of medical instruments is critical in resource limited settings if disposable syringe or medical consumable products are not available [34, 35]. Medical behavior should be monitored strictly to ensure high quality services and lower unprotected exposure to clinical HIV or HCV infections. Proper management of HIV-HCV co-infection is critical and can be highly effective in disease control [36]. Prevention and control strategies should be implemented comprehensively to ensure their maximal effectiveness.
